# The feasibility and acceptability of a task-shifted intervention for perinatal depression among women living with HIV in Malawi: a qualitative analysis

**DOI:** 10.1186/s12888-022-04476-z

**Published:** 2022-12-29

**Authors:** Kazione Kulisewa, Josée M. Dussault, Bradley N. Gaynes, Mina C. Hosseinipour, Vivian F. Go, Anna Kutengule, Katherine LeMasters, Samantha Meltzer-Brody, Dalitso Midiani, Steven M. Mphonda, Michael Udedi, Brian W. Pence, Angela M. Bengtson

**Affiliations:** 1 Department of Psychiatry and Mental Health, Kamuzu University of Health Sciences, Faculty of Medicine, Private Bag 360, Blantyre, Malawi; 2 Department of Epidemiology, Gillings School of Global Public Health, University of North Carolina at Chapel Hill, Chapel Hill, NC USA; 3 Department of Psychiatry, UNC School of Medicine, University of North Carolina at Chapel Hill, Chapel Hill, NC USA; 4 UNC-Project Malawi, Lilongwe, Malawi; 5 Department of Medicine, UNC School of Medicine, University of North Carolina at Chapel Hill, Chapel Hill, NC USA; 6 Department of Health Behavior, Gillings School of Global Public Health, University of North Carolina at Chapel Hill, Chapel Hill, NC USA; 7 Ministry of Health, Lilongwe, Malawi; 8 Department of Epidemiology, Brown School of Public Health, Brown University, Providence, Rhode Island USA

**Keywords:** Perinatal depression, Mental health, HIV, Malawi, Task-shifting

## Abstract

**Background:**

Perinatal depression (PND) is prevalent and negatively impacts HIV care among women living with HIV (WLHIV), yet PND remains under-identified in Malawian WLHIV. Accordingly, this formative study explored perceptions of the feasibility and acceptability of an integrated, task-shifted approach to PND screening and treatment in maternity clinics.

**Methods:**

We completed consecutive PND screenings of HIV+ women attending pre- or post-natal appointments at 5 clinics in Lilongwe district, Malawi. We conducted in-depth interviews with the first 4-5 women presenting with PND per site (*n* = 24 total) from July to August 2018. PND classification was based on a score ≥ 10 on the Edinburgh Postnatal Depression Scale (EPDS). We conducted 10 additional in-depth interviews with HIV and mental health providers at the 5 clinics.

**Results:**

Most participants endorsed the feasibility of integrated PND screening, as they believed that PND had potential for significant morbidity. Among providers, identified barriers to screening were negative staff attitudes toward additional work, inadequate staffing numbers and time constraints. Suggested solutions to barriers were health worker training, supervision, and a brief screening tool. Patient-centered counselling strategies were favored over medication by WLHIV as the acceptable treatment of choice, with providers supporting the role of medication to be restricted to severe depression. Providers identified nurses as the most suitable health workers to deliver task-shifted interventions and emphasized further training as a requirement to ensure successful task shifting.

**Conclusion:**

Improving PND in a simple, task-shifted intervention is essential for supporting mental health among women with PND and HIV. Our results suggest that an effective PND intervention for this population should include a brief, streamlined PND screening questionnaire and individualized counselling for those who have PND, with supplemental support groups and depression medication readily available. These study results support the development of a PND intervention to address the gap in treatment of PND and HIV among WLHIV in Malawi.

**Supplementary Information:**

The online version contains supplementary material available at 10.1186/s12888-022-04476-z.

## Background

Depressive disorders during pregnancy and the first year after delivery are estimated to affect 11.3 – 18.3% of all pregnancies [1], with the burden of perinatal depression (PND) in low and middle income countries (LMIC) consistently being reported as greater [2, 3]. Among African women living with HIV (WLHIV), the prevalence of PND is much higher, estimated to range from 22.5 - 43.5% [4]. PND poses a substantial public health burden [5, 6] and has been implicated in adverse pregnancy outcomes such as poor fetal development, preterm deliveries and low birth weight [7, 8]. PND has a negative impact on maternal health, maternal quality of life and increases risk of maternal suicide [9, 10]. PND also adversely impacts mother-infant attachment [5], increases the risk of infant malnutrition [11] and contributes to poor cognitive development in infants [5, 6]. The effects of PND extend beyond the mother-infant dyad, with PND increasingly being recognized as a risk factor for depression and psychiatric disorders in partners and children [12, 13]. In WLHIV, PND may have a negative impact on the HIV care continuum as untreated depression is associated with poor linkage to care, decreased antiretroviral treatment (ART) adherence and poor viral suppression [14–17]. As a detectable viral load increases the risk of virus transmission, PND may subsequently also impact mother to child HIV transmission.

Malawi has made progress in addressing the HIV pandemic: in 2018, an estimated 90% of the one million individuals living with HIV were aware of their status, 78% were on treatment and 69% were virally suppressed [18]. However, young women remain a key population, with the rate of new infections in this demographic equalling more than double that of their male counterparts [18]. The antenatal period represents a unique opportunity for WLHIV to be linked to HIV care services [15] with more than 95% of pregnant WLHIV accessing ART. However, one in five postnatal WLHIV are lost to follow up within 6 months of ART initiation [19–21]. Untreated depression, with its documented association with poor linkage to care [15–17] and poor ART adherence [22], may play a role in the high rates of poor engagement in postnatal HIV care. Limited data exist exploring the prevalence and impact of PND among Malawian mothers [23]. Among women not living with HIV, the available data estimate that 10.7-14.2% of pregnant Malawian women suffer from antenatal depression [24, 25]. While a sole Malawian study estimates a similar PND prevalence in perinatal WLHIV [23], regional sub-Saharan studies suggests that the prevalence of PND in WLHIV is much higher [4, 6, 26, 27].

Much of this burden of PND likely remains under-recognized and untreated in Malawi, with depression management strategies either under-developed or under-utilized. For example, the Edinburgh Postnatal Depression Scale (EPDS)—a depression screening tool with widespread use [28, 29]—was translated, adapted and validated in Malawi in 2012 [30]. However, its use in screening for PND remains limited to a few research settings. Various psychosocial interventions developed in sub-Saharan Africa, such as individual or group problem-solving therapy, have shown efficacy in managing common mental disorders in people living with HIV [31–34], but are yet to be translated into routine and widespread practice in Malawi. Similarly, antidepressants, stated as available in the country’s essential health package [35], are largely utilised only in tertiary mental health settings and not in routine antenatal or postpartum care. Factors that have contributed to the poor uptake of these efficacious interventions in maternity care settings are yet to be fully elucidated. Given the high burden of undiagnosed PND and its implications for HIV care, knowledge of the factors that influence the uptake of depression interventions may be critical in achieving the UNAIDS goals of ensuring high patient retention throughout the HIV care continuum [36] in perinatal populations.

Aside from the under-utilization of depression interventions in perinatal care delivery, Malawi is further constrained by having limited mental health personnel to deliver the interventions. Mobile health (mHealth) interventions have shown promise as solutions to improve maternal health in human resource limited LMIC [37]. However, the current Malawian landscape limits the potential utility of mHealth interventions: phones are disproportionately owned by men; few women possess smart phones, thereby limiting any potential interventions to short message service (SMS) platforms; illiteracy among women also challenges the routine use of SMS as interventions [38]. An alternative approach for addressing inadequate specialised personnel is task-shifting. As many of the aforementioned depression interventions can be task-shifted and integrated into existing healthcare systems [31, 32, 34], this strategy has particular appeal for the Malawian setting. We report here on formative research to understand the perceptions of health providers and WLHIV about the feasibility and acceptability of task-shifted PND screening and treatment interventions, integrated into routine perinatal care. These data have supported the subsequent development of an integrated PND intervention.

## Methods

### Study objectives

Our goal was to understand the feasibility and acceptability of integrating task-shifted depression screening interventions into routine maternity (antenatal and postnatal) care. To accomplish this, we conducted semi-structured individual interviews with WLHIV and their providers. These interviews also explored acceptable and feasible therapeutic intervention strategies for managing PND among WLHIV, including the potential for these treatments to be delivered by non-mental health providers at the facilities.

### Study setting, population and sample

#### General setting

Although pregnant women in Malawi are encouraged to attend maternity services early in the first trimester, more than half present in the fourth month or later for their first antenatal visit [39] and few achieve the World Health Organization recommended minimum of four visits [40]. Antenatal women are routinely tested for HIV with guidelines recommending women testing positive for HIV are immediately initiated on ART. HIV care and antenatal care are integrated, with ART being dispensed at routine antenatal visits. After the puerperal period (approximately six to 8 weeks postpartum), mothers living with HIV are transitioned to adult ART clinics for ongoing HIV care sometime in the first year postpartum.

In Malawi, integrated HIV and antenatal/postnatal services at primary health centers are largely provided by nurses who typically have midwifery training. Another cadre of health worker involved in the delivery of maternity services are clinicians (‘clinical officers’) who have 3 years of medical training.

The knowledge, exposure, and experience of these nurses and clinicians to mental health care is variable. Staff designated as nurse-midwives typically have 6 weeks of mental health teaching during the three (Nursing diploma) or four (Nursing degree) years of nurse training. Qualified nurses can further specialise through a one-year certificate in mental health nursing and are designated Mental Health or Psychiatric Nurses. The mental health curriculum, during the three-year medical training clinical officers undergo, consists of 2 weeks of theoretical teaching and a further 2 weeks of clinical experience. The lack of integration of mental health in the Malawi public health system implies most primary care providers have limited professional experience in handling mental health conditions. While some nurses may have a nominal mental health designation, they are frequently involved in other aspects of health care delivery including midwifery, perinatal care, and ART services. All health workers who provided maternity care at the five sites were eligible for participation in this study.

#### Study sample

The study was conducted at five maternity clinics in Lilongwe district, Malawi. The maternity clinics were based at two urban/peri-urban primary health care centers and three rural primary health care centers. Qualitative interviews were conducted between July and August 2018. All WLHIV seeking prenatal or postnatal care at the study sites during the days of recruitment were informed about the study while they were in the waiting room. They were consecutively invited to complete the EPDS with a trained psychosocial counsellor. Before beginning the EPDS, participants were required to provide their written informed consent. WLHIV who screened positive for PND (EPDS score ≥ 10) and who were over the age of 18 were invited to complete an in-depth interview with a trained research assistant. Women who endorsed self-harming thoughts on the EPDS underwent a second screening with a suicide risk assessment protocol to assess the severity of any potential suicidal ideation. Individuals with active suicidal ideation were subsequently referred to specialist mental health services for further assessment.

Records were not maintained on daily clinic attendance, and we were therefore unable to estimate the proportion of clinic attendees who consented to be screened with the EPDS. However, studies have shown that sensitization (i.e., providing information about the study before inviting participation) can result in high levels of voluntary participation [41, 42]. In total, 73 perinatal WLHIV consented to complete the depression screening across the five clinics, and 24 (33%) WLHIV scored 10 or greater on the EPDS. These 24 WLHIV subsequently participated in in-depth interviews. We also conducted 10 in-depth interviews with maternity care providers using a convenience sample of two providers per site.

### Data collection process and tools

The EPDS is a 10-item screening tool validated to detect probable PND during both the antenatal and postnatal period [23, 24, 43]. The EPDS has been translated into Chichewa, the local language in Malawi, and adapted for a low-literacy population through the use of pictures and administration by a health worker [30]. The 10 items are individually scored to provide a total score. Total scores of ≤9 are suggestive of short-lived symptoms that are unlikely to meet the threshold for a diagnosis of depressive or anxiety disorders; scores of 10-12 suggest distressing emotional symptoms that potentially impair functioning, and cut-off scores exceeding 13 are highly predictive of depressive or anxiety disorders [43]. We defined PND as EPDS total scores of ≥10 [30]. The administration of the EPDS by a study researcher, an experienced metal health nurse, required 10-15 minutes depending on the participants’ comprehension of the tool’s questions.

In-depth interviews utilized semi-structured guides whose questions were informed by the previously described goals of this study using a deductive and iterative process (Additional files 1 & 2). The guide for WLHIV prompted topics such as lived experiences of PND; the possible impact of PND; the perceived need for PND screening; and women’s attitudes towards PND treatment modalities, including counselling and antidepressant medication. Similarly, the providers’ guide addressed providers’ previous experiences with patients who had PND and the feasibility and acceptability of screening and various treatment interventions for PND integrated into routine maternity HIV care. All in-depth interviews were conducted by one female Malawian researcher (AK) and were largely conducted in Chichewa, with a few health workers interviewed in English. Prior to conducting interviews, AK attended a 3-day training led by study team members with expertise in qualitative methods—the training discussed the iterative model of qualitative research, rapport-building, the flexibility of discussion provided by the semi-structured interview guide, and how to probe for more information or clarity. Interviews were conducted individually at the facilities using private and confidential rooms.

### Data analysis

Interviews were recorded on audiotapes and were simultaneously translated and transcribed into English by the interviewer (AK). Informal analysis occurred simultaneously with data collection to facilitate the exploration of further topics. Transcripts were uploaded to NVivo 12 for qualitative analysis [44]. Members of the study team met frequently to iteratively develop and modify the codebook using a general inductive approach: while initial codes were generated based on the interview guides’ structure, the eventual codebook was revised and refined based on the structure and themes of interview transcripts [45]. A primary coder (JMD; American based in the U.S.) was responsible for coding all interview transcripts, and they met regularly with the original interviewer (AK) and other research team members to clarify contextual questions and refine the codebook. Once a stable codebook was established, two study members (JMD and KL) coded the same five interviews, varying in interviewee demographics, to test the codebook’s reliability. This process revealed high codebook reliability, and the remaining interviews were then coded by the primary coder (JMD). Qualitative codes were then summarized by developing matrices to identify the case attributes associated with particular codes and sentiments [46]. These matrices were interpreted and discussed between JMD and KK, a Malawian psychiatrist, along with other members of the study team. The summarized results, paired with illustrative quotes, are presented here.

### Ethical approval

This study was approved by the Institutional Review Boards at the University of North Carolina at Chapel Hill and at the Malawi National Health Sciences Research Committee.

## Results

### Sample characteristics (Table 1)

The study sample consisted of 24 WLHIV and 10 health workers. The median age of WLHIV was 29 years. 54% of the WLHIV were interviewed in the prenatal stage. Although most women (62.5%) had previously been diagnosed with HIV, none had previously been identified as having comorbid PND and therefore had never received any mental health interventions. Among the 24 WLHIV, suicidal ideation was reported in 14 (58%) participants. Nine of these participants were assessed to have passive suicidal ideation and five had active suicidal ideation. Except for counselling treatment preferences, participants’ responses did not vary by suicidal ideation severity. Health worker participants were typically female and designated as HIV care (ART) providers; only two health workers were specialized in mental health.

**Table 1:** Participant characteristics (*N* = 34)

**WLHIV (*n*** **= 24)**	**Count (%)**
** Median (IQR) Age of WLHIV, years**	29 (24, 35)
Median (IQR) EPDS Score	15 (12, 21)
** Pregnancy stage**	
Prenatal	13 (54.2)
Postnatal	11 (45.8)
** Number of pregnancies**	
1	3 (12.5)
2	9 (37.5)
3	6 (25.0)
4	4 (16.7)
5+	2 (8.3)
** HIV diagnosis**	
Current pregnancy	9 (37.5)
Previously diagnosed	15 (62.5)
** Perinatal depression diagnosis**	
Previously diagnosed	0 (0.0)
Current study	24 (100.0)
** Suicidal Ideation Severity**	
None	10 (41.7)
Passive	9 (37.5)
Active	5 (20.8)
**PROVIDERS (*n*** **= 10)**	
** Gender**	
Male	1 (10.0)
Female	9 (90.0)
** Nominal Designation**	
Clinic Director	1 (10.0)
HIV care provider	7 (70.0)
Mental health nurse	2 (20.0)

### Feasibility of integrating PND screening into routine HIV antenatal care

The feasibility of integrating PND screening into the existing antenatal and HIV care was evaluated using descriptive codes that identified facilitators and barriers for the intervention from the perspectives of providers and WLHIV. For identified barriers, participants also reported possible strategies that could be used to address the barriers.

#### Facilitating attitudes to integrated PND screening

PND screening was universally welcomed by perinatal participants with WLHIV reporting that they wished to know their mental health status. One WLHIV reported, *“I would welcome it [PND screening]. I would know that they [health workers] want to help us in some way, for us not to be depressed. Depression can bring about so many problems and even taking your own life”* (35-year-old prenatal patient).

The semi-structured interviews also assessed provider attitudes towards integrated screening for PND. With the EPDS in mind, the interviewer asked the providers individually their views on how feasible it would be to integrate an unspecified, structured 10-item depression screening questionnaire into their routine day-to-day care of perinatal women. Eight out of 10 providers across the five sites endorsed the feasibility of integrating PND screening into perinatal care. Some providers suggested they would value PND screening, as they appreciated that untreated depression has a potentially severe impact on maternal and child health: *“[A depression screening questionnaire] would be a very good thing, because we would know which patients are depressed in good time ...depression can get severe, and so before it reaches that point, we would know that a person is depressed”* (Antenatal Nurse).

A provider further endorsed the intrinsic value of screening for depression stating that any holistic perinatal care package must have a mental health component: *“Every woman has to be screened for such conditions. … Our maternal admission sheet also has a component of asking about the psychiatric history, but isn’t comprehensive, so if we can integrate this into that... then it can be feasible”* (Clinician). The provider suggested integrated depression screening would be feasible as an existing foundation was present in the current maternal admission sheet which could be built on.

Health workers suggested that during the postnatal period they were already attuned to possible psychosocial problems or emotional problems in new mothers: *“We’re supposed to make sure that she is fine before sending her home …*. *She may look unhappy as she responds to the questions. … we notice more during the post-natal period than antenatal”* (Mental Health Nurse). The provider however reported that health workers lacked a standardised method of detecting PND, a structured method for screening would therefore be welcomed: “*… But there is no procedure or guide for screening if the patient is depressed or if she is just sad with something.”*

#### Barriers to integration and possible solutions

Although PND screening was welcomed by WLHIV, one woman suggested that the community may not value the intervention as much as depressed patients would: *“People would be like, ‘they want to test if I am depressed? Why … would they test me for that?’ If it’s a problem for people to accept the blood transfusion, … what more testing for depression? As for me, I would accept”* (27-year-old prenatal patient). The WLHIV suggested that community understanding of depression was poor and this lack of knowledge could be a potential barrier.

Providers identified that certain health workers would possibly not welcome depression screening as this would be perceived as additional work on staff who were already over-burdened with multiple provider roles. A health worker reported *“… What happens most of the time is that, when new things are being introduced, we feel like they are just adding on to our work. So those are the things that bring about a negative attitude”* (ART Nurse). The health worker, reflecting the views of other providers, further emphasized that additional, related barriers to the integration of depression were logistical barriers of inadequate human resources and the time required to conduct a comprehensive depression screening: *“Sometimes it’s because of our staffing levels. I’ve been working alone since I came in this morning, I’ve been providing family planning and antenatal services. Today we had those two HIV-positive women and … to do the depression screening (laughs). … That could make it challenging.”*

Providers suggested that the facility barriers could possibly be overcome through the introduction of extra staff to conduct the PND interventions. Other providers noted that these barriers could be overcome through staff training to improve knowledge on the importance of PND screening: *“People must understand [depression screening], because the moment people don’t understand...then we are bringing the issue of resistance. Before we introduce this screening tool, people have to be taught and have to be explained to, the essence of screening for depression”* (Clinician). The provider also stated that *“These screening tools should be... as short as possible, maybe with a few questions … Make sure that the screening tool is not that difficult to fill... to go through,”* suggesting that any intended screening tools be adapted to create a modified brief tool that was shorter and easy to use.

One provider (a clinician) also suggested that regular supervision from program coordinators would play an important role in ensuring the facility successfully implemented the intervention:

*The supervision is very important in everything, because you can do the training but when you get on the job you find that you have forgotten some of the things, you are skipping some things...but under supervision you fix some other areas. You know the steps to follow so when you are supervised, work is done well. It could be monthly, no problem. When you are being mentored, things go well.*

### Treatment preferences

The acceptability of two broad modalities of depression treatment—antidepressant treatment and counselling therapy—were explored by assessing participant preferences. In discussing counselling, WLHIV further expressed the merits and disadvantages of individual counselling, group counselling and pastoral counselling.

#### Patient-centred counselling

The interviewer explored the WLHIV’s personal experience of perinatal depression or probed to gauge the women’s understanding of the condition. Among these probes were the women’s perceptions on ways for the PND to be addressed. In their narratives, many women spontaneously expressed the importance of being able to talk to someone, frequently citing the need to unburden themselves. One WLHIV described her preferred approach to treating a woman with PND: *“I would chat with her frequently, so that her depression goes away, and I would encourage her, so that she gets strengthened in her heart”* (21-year-old prenatal woman).

When talking was suggested as therapeutic by WLHIV, the interviewer introduced counselling as possible intervention at the facility. Most WLHIV (88%) welcomed counselling as an intervention to treat PND. Only two WLHIV expressed ambivalence about counselling in general as a modality for treating depression. A woman supportive of counselling stated the following:

*“I would be okay with [counselling] because people have so many worries in the homes. They don’t know where to take their worries to and as a result people contemplate buying poison to take their own lives, or they just cry in their rooms and are sad. They refuse to eat, and, because of that, they begin to lose weight, etc., because of not eating, so you need to chat with that person. She gets helped and the depression may reduce”* (24-year prenatal patient).

Providers similarly identified counselling as the preferred first line therapy for PND, especially if the depression was mild: *“It depends on the severity of depression, when we see a woman who is depressed, … first of all, we have to assess the severity: is it mild, moderate or severe. For mild forms, we may come in with maybe counselling”* (Clinician). Providers further emphasized that acceptable and effective counselling must be tailored to a patient’s needs, exemplified in the words of this postnatal care nurse:

*The patient shouldn’t be dictated on what to do. Because how I can cope with a situation I find myself in is different from the way you would, … so the counsellor should be accommodative and involve the client. For that to happen, you need a conducive environment and also good [sufficient] time so that it is not done in a hurry. It should happen in an isolated place, the patient should be involved, and there should be enough time. Sometimes the person may not believe you, but as time passes, she develops a trust for you and starts to open up. That’s what I think.*

#### Individual counselling versus group counselling

WLHIV were further asked to qualify their specific preferences on the type of counselling, and 22 of 24 women expressed their preferences. These preferences varied, and the presence of suicidal ideation in participants was largely associated with a preference for group counselling: of the 10 participants with no suicidal ideation, seven (70%) expressed a preference for individual counselling, while three (30%) preferred group counselling. By contrast, only two of the nine (22%) women with passive suicidal ideation expressly preferred individual counselling, while six others (66%) expressly preferred group counselling, and one (11%) was equally pleased with both options. Among the five women with active suicidal ideation, only one (20%) voiced a preference for individual counselling, while two (40%) more preferred group counselling and two (40%) did not respond to the prompt.

WLHIV who preferred individual counselling stated that they felt more comfortable with disclosure to a hospital therapist and reported concerns about poor confidentiality in group therapy. A 26-year-old postnatal patient noted:

*I would choose to come individually because there are some things I can’t say in a group. What would happen is that I would not be talking in the group. I would just be listening. I wouldn’t share all my worries. I would hide some things. Especially my marital issues. Some of my neighbours could be part of the group so I would be afraid of being laughed at back home so I wouldn’t share everything in the group. I am able to tell you everything because it is just the two of us in here and I know you won’t tell anyone, and you work at the hospital. It’s different from having my friends in the same club or group. That’s how they can find an opportunity to talk about me*.

While there were very clear concerns about confidentiality in a group setting, other participants saw its benefits. One woman who supported group counselling stated, *“I would be so happy about [group counselling]. I would be freed; my depression would end, and I wouldn’t act on the bad thoughts that I have”* (33-year-old prenatal patient). This patient suggested that peer support was an important merit of group therapy: *“I would be happy if we were to do it with women going through similar things because we would help each other out. However, there is need for someone else to guide us since we are all depressed and it is difficult for us to help each other.”*

#### Pastoral counselling

Several WLHIV (*n* = 9) suggested that religion and pastoral counselling could play an adjunctive role alongside treatment received at the hospital: *“Religious people encourage you from religious point of view that you shouldn’t rely on drugs only, but you should also be praying, and they encourage you to do both”* (35-year postnatal patient). However, other WLHIV had negative views regarding pastoral counselling. One such woman stated, *“I wouldn’t use it … they [pastors] don’t understand those of us who have HIV”* (21-year prenatal patient). The woman expressed that religion was sometimes used in conflicting ways to hospital-based care which could predispose patients to poor clinical outcomes: *“They can tell you that they have prayed for you, and you will be fine, and they tell you to throw the medicine in the toilet. If you really do that you can get in trouble and die.”* While most providers (*n* = 6) also endorsed the possible role of pastoral counselling, three providers expressed reservations and suggested that religious leaders need to be regulated in the context of providing support to patients.

#### Antidepressant medication

WLHIV participants were largely unaware of the existence of antidepressant medications which could effectively treat depression: *“I don’t know of the existence of depression medication. Medicine? To remove someone’s depression, I have never heard of such a thing”* (27-year prenatal patient). In the context of the low levels of knowledge on the role of medication in treating depression, women generally had ambivalent or negative attitudes towards the use of medication during pregnancy. Concerns that were cited included additional pill burden, fears over the teratogenic potential of medications and community perceptions of women who took medication during a pregnancy. One WLHIV expressed, *“When you are pregnant and you take meds, some people think that you want to abort the pregnancy. Yes, they say you want to abort the pregnancy”* (20-year postnatal patient)*.* However, attitudes towards medication became more permissive after the interviewer explained the efficacy of antidepressant medications, and all 24 WLHIV participants subsequently agreed that they would use antidepressants if prescribed.

Unlike counselling, medication as a treatment option was not spontaneously suggested by most providers. The interviewer suggested medication as an alternative therapy and asked providers to discuss their views on the use of antidepressants. In contrast to patients, health workers immediately endorsed the efficacy of antidepressants in treating PND. However, the general view of health workers was that antidepressant use was indicated in cases of severe depression while mild depressive symptoms were best managed through counselling. Reflecting her background in mental health training, one mental health nurse stated, *“The depression medications that we have that help: there’s amitriptyline and fluoxetine. These are the medications that are used for women who are depressed.”* She further reported, *“Some patients, they only get depressed for a short period of time. They just need someone to help them with counselling and some companionship... For others, though, the depression can continue for up to 6 weeks. Those ones need treatment [medication]”* (Mental health nurse).

While the mental health nurse demonstrated some familiarity with antidepressant medication, some providers suggested this should be an element of any subsequent training. An antenatal nurse presented the following suggestion:

*The people need to be trained on the appropriate medication to administer to someone who is depressed … After training, we could be given a period of 3 months and they can come again to supervise if we are able to do what we were trained in. You don’t get everything when you go for training. You miss out on some things, and so if the person comes for supervision, it’s like she reminds you of what you learned.*

### Feasibility of health workers delivering screening and treatment interventions in a task-shifted model

Providers were asked several open-ended questions on which group of professionals at their facility would be best equipped to deliver the screening and treatment interventions for depression in a task-shifted model. Providers designated nurses as the most suitable cadre of health workers to provide the screening and counselling intervention because nurses have the most frequent contact with patients. One ART nurse explained:

*… Because the clients are usually in contact with nurses. The doctor just comes in here and there when they are called. The patient is mostly in contact with a nurse like during pregnancy, it is only a few women who have major problems that require a doctor or clinician’s attention. Most of them complete their antenatal visits and deliver without ever meeting a clinician. The patients spend more time with nurses than anyone else.*

Providers stressed the importance of further training to enable them to deliver any potential mental health treatments: *“The knowledge of depression that people have is from classes they took long ago, some have forgotten the stuff”* (Mental health Nurse). This same provider suggested that trainings should have content on aetiology, presentation, and treatment of PND: *“They [providers] need to go for training to learn what perinatal depression is … and how a woman looks when she is experiencing it and how they can help such women.”*

## Discussion

This study explored the perspectives of health workers and WLHIV on developing an integrated task-shifted intervention to address PND in WLHIV. The proposed task-shifted intervention for PND had two facets: a structured screening assessment tool that could be integrated into routine perinatal care, and a treatment intervention to be delivered by the provider. WLHIV welcomed PND screening, as did most providers. Providers who endorsed the feasibility of integrating depression screening as routine care stated they appreciated the potential heavy morbidity associated with depressive disorders and felt the introduction of PND screening would be addressing a current gap in the provision of maternity care. Providers however suggested that negative health worker attitudes to innovations, inadequate staffing levels and time constraints could prove barriers to the provision of PND screening. Counselling was identified as an acceptable and preferred treatment option. However, the participating WLHIV had divergent views on the role of individual counselling or group counselling, citing the merits and weaknesses of both counselling formats. There was also low patient knowledge on the safety and efficacy of antidepressant medications, with WLHIV expressing that there was a potential for community stigma if they were seen taking medications during pregnancy. Health workers suggested that the role of antidepressants should be reserved for severe depressive illnesses. Health workers endorsed nurses as the cadre of providers best suited to screen and treat PND using counselling or antidepressants at their facilities and emphasized the need for additional training for task-shifting to be successfully implemented.

Task-shifting is increasingly being advocated in LMIC to bridge the significant mental health treatment gap [47, 48]: the under-provision of care for PND in perinatal WLHIV is an example of such a treatment gap. However, task-shifting approaches are frequently assumptive about the capacity of primary health workers to deliver mental health interventions [49]. While task-shifting of mental health has demonstrated success [48], primary health workers in Malawi already perform many roles as exemplified by the nurse who described playing the role of a family planning, antenatal care and ART provider. Attempting to further integrate mental health care among the many other roles these nurses perform may potentially be met with resistance. In considering PND screening, providers identified several challenges, with negative attitudes among staff recognised as a prominent barrier to the integration of the intervention. Implementation approaches should therefore have specific strategies to overcome such threats to task shifting.

In their systematic review of barriers and strategies to the implementation of clinical guidelines, Fischer et al. (2016) report that negative staff attitudes are best challenged by having opinion leaders endorse the innovation [50]. Individualised audit and feedback are also stated to be instrumental in improving motivation among health workers [50]. As suggested by several nurses in the current study, Fischer et al. also identify that the lack of familiarity with interventions limits the uptake and use of such interventions [50]. Effective implementation must therefore employ tailored strategies such as disseminating educational materials, visibly displaying materials in consultation rooms, and encouraging continuous medical education to improve health worker knowledge [50]. Consideration of the barriers we identified suggests that health workers at the sites must be trained on the aetiology, manifestation, impact, and management of PND. As opinion leaders, the endorsement and involvement of clinic leadership in these processes will be essential. The motivation of health workers to integrate PND screening into their routine practice could similarly be enhanced through regular supervision and auditing health worker performance on PND screening.

Aside from personal factors among health workers, interventions may have inherent structural factors that may limit their use: extensive tools and tool complexity are two such barriers [50]. The widespread use of the EPDS in various primary healthcare settings by non-mental health staff [28, 29] suggests that this 10-item screening questionnaire may meet the requisites of health workers who requested a user-friendly screening tool that is “… not that difficult to fill in …” However, a further consideration before employing the EPDS is whether the 15-20-minute completion time would be considered acceptable by busy providers who require that the screening be “… as short as possible.” The Self Reporting Questionnaire 20-Item (SRQ-20) has also been used in perinatal research settings in Malawi with comparable validity to the EPDS [25, 30]. Although it is a 20-item questionnaire, the dichotomous “yes” or “no” nature of responses required by the SRQ-20 may mean this is a quicker and easier tool for health workers. Health worker preferences in respect to these two tools need to be further explored.

Time constraints and heavy workload are genuine challenges in the context of primary healthcare in Malawi that require careful consideration and solutions. The financial incentives for health workers—suggested by Fischer et al. [50]—may not be a sustainable solution to these organisational constraints for the Malawian setting. Nevertheless, this qualitative analysis has identified other viable alternatives to facilitate PND screening and treatment in a human-resource limited environment. These alternatives include: 1) developing a brief screening procedure that facilitates rapid depression screening while folding into providers’ existing workflows; and 2) generating an implementation strategy that focuses on provider education, facility leadership support, and supervision of screening practices. Clear provider role definition and the development of organisation-specific protocols may further enhance the feasibility of PND screening at these facilities [50].

Various task-shifted counselling approaches have demonstrated success in treating depression [31, 32, 51]. Notably, an intervention developed by Nyatsanza et al. (2016) for managing PND in an urban South African community may serve as a useful framework for the development of a similar intervention in our population: although not in a population living with HIV exclusively, formative work conducted prior to the development of that intervention suggested that a clinic-based counselling intervention would be acceptable to most patients if delivered by a middle-aged woman who spoke the local language fluently [52]. As midwives expressed some resistance to taking on additional mental health roles, Nyatsanza et al. concluded that community health workers would be the most suitable cadre to deliver the intervention. These health workers received a five-day training in counselling and were subsequently supported through regular group and individual supervision and debriefing. They delivered a form of counselling that combined problem solving and cognitive behavioural approaches and was manualised into a highly structured, six-session intervention. The first session focused on psychoeducation for depression and the second session utilised problem-solving skills to deal with common problems. Subsequent sessions respectively addressed behaviour activation, maladaptive behaviours which exacerbate depression, healthy thinking to target cognitive distortions, and preparation to resolve anxiety around birth. The final session evaluates content learned over prior sessions [52].

Although the subsequent randomized trial that implemented the intervention described by Nyatsanza et al. (2016) did not find a significant improvement in PND after the intervention [52, 53], Lund et al. (2020) theorized that the intervention was likely deficient in some key ways: First, they theorised that six therapy sessions may have been an inadequate dose and could not surmount the adverse social and economic circumstances of the women with PND. The content of the therapy may also have been unsuitably complex relative to the short number of sessions. Moreover, only 53% of the participants actually completed all six sessions [53]. And finally, intervention delivery fidelity was decent (62.8%), but Lund et al. theorized that fidelity could have been improved by modifying the duration or quality of the training and supervision that the community health workers received [53]. Therefore, while task-shifted PND interventions have promise, there must be careful attention paid to ensure that the cadre of health workers intended to deliver the intervention is appropriate, the intervention complexity matches the training of the providers, and the intervention content is suitable for the target population.

In the current study, perinatal WLHIV demonstrated poor knowledge on the efficacy of antidepressant medication and, while they frequently emphasised the psychosocial aspects of depression, they appeared to be unaware of the biological determinants. This suggests that a locally relevant depression intervention must include a significant psychoeducation component to improve the mental health literacy of perinatal women. Initial counselling sessions must impart knowledge on the various modalities of depression treatment, including the benefits and potential risks of antidepressants, allowing women to make informed choices and actively participate in their own treatment. Initial sessions must also focus on rapport building, as a recurring theme in the interviews was women’s fear of the violation of their confidentiality. The counselling intervention must invest in creating rapport between counsellor and patient and re-affirm the absolute confidentiality of the counsellor-patient relationship, thereby allaying fears of violation of privacy. Only in such an environment will women be confident in their disclosure.

Our cohort of women participants had differing views on the role of individual counselling or group counselling. Taking into consideration the stated fears of confidentiality violation, a possible approach would be individual counselling with supplemental group therapy as an option for WLHIV. Modelling on the intervention described by Nyatsanza et al., individualised counselling would be structured over several sessions, although attendance may foreseeably be challenged by late presentation for antenatal services and limited number of antenatal visits, which are both common in the antenatal care system in Malawi [39, 40]. As suggested by one of the interviewed providers, any therapeutic approach would need to be patient-centred and guided by patient requirements. Due to this prerequisite, the Friendship Bench intervention, developed by Chibanda et al. [33, 34], would have appeal as an acceptable potential therapeutic intervention for perinatal WLHIV. The Friendship Bench is an evidence-based individualised counselling protocol developed in Zimbabwe based on problem-solving therapy. Counselling sessions are patient-centred with the focus on priority problems identified by the patient. The counsellor guides the patient to the formulation of a specific, practical, and actionable plan to target the problem. The intervention uses task-shifting approaches: various trials of the intervention have used different lay health workers to deliver the counselling and effectively manage common mental disorders and depressive symptoms in populations living with HIV [33, 34].

Most providers in this study nominated nurses as the ideal cadre of health workers to deliver mental health counselling to WLHIV due to their frequent contact with patients, both in the antenatal and postnatal period. However, the multiple roles that nurses already occupy in perinatal care raise doubt on whether nurses can sustainably perform this added function. Perhaps other cadres of health workers, including lay health workers, should be considered as providers of perinatal mental health interventions. For example, the providers who participated in this study frequently described time constraints and heavy workload as barriers to implementing PND screening alone. Thus, one could anticipate that the regular delivery of 45-minute counselling sessions (a typical requirement for structured counselling approaches such as the Friendship Bench) would not be sustainable within nurses’ already heavy workloads. As described above, some counselling-based mental health interventions are amenable to being delivered by lay health workers or community members with minimal medical training [32, 33]. Any approach utilising lay health workers must nonetheless address the aforementioned challenges that undermine the effectiveness of task-shifted PND psychosocial interventions [53]. Further research is therefore required to investigate the capacity and motivation of nurses to deliver the counselling in our study population or alternatively identify other hospital staff, lay health workers, or peers who would be more suited to deliver counselling.

### Limitations

As the study was hospital-based, with respondents being interviewed by an individual who may have been perceived to be affiliated with the institution, the responses of our participants may have been influenced by a degree of social desirability bias: health workers may have readily assented to the integration of PND screening and counselling as feasible, even if it conflicted with their true beliefs, to avoid being labelled as resistant. Similarly, WLHIV may have reported that interventions such as counselling were acceptable in anticipation of a favourable response from the interviewer. A further possible limitation of the study was the small sample size, however the convergence of participants’ views on the discussed topics suggests that saturation was achieved, and the sample was adequate. This assumption is supported by research that has demonstrated that qualitative studies within relatively homogenous study populations are capable of reaching saturation in as few as nine individualized interviews [54]. The generalizability of the study findings may also be limited as the study was conducted in a single district within Malawi. However, the distribution of participants across urban, peri-urban, and rural maternity clinics may improve the generalizability.

## Conclusion

Task-shifting approaches have the potential to address the treatment gap for perinatal WLHIV requiring mental healthcare in LMIC. Successful task-shifting approaches must however identify and address potential barriers to be effective. This study of Malawian women living with HIV and PND and their healthcare providers highlighted the need for a sustainable PND intervention to be integrated into perinatal care. While barriers are present in the Malawian landscape, providers suggested such integrated screening for PND could be feasibly implemented with a protocol that is modified to meet time and personnel constraints. WLHIV required increased knowledge about depression and mental health, and any relevant treatment intervention should have a significant psychoeducation component. WLHIV also readily endorsed the acceptability of counselling therapies, therefore various task-shifted counselling interventions that have demonstrated efficacy in similar populations have a strong potential to meet the mental health treatment needs of this vulnerable population, and consequently improve health of mothers and their families. Provider training in PND detection and care, and subsequent supervision, will be crucial in the successful implementation of any task-shifted intervention.

## Supplementary Information

**Additional file 1. **Perinatal Depression and HIV – Provider’s Interview Guide.

**Additional file 2. **Perinatal Depression and HIV - Patient Interview Guide.

## Data Availability

The data generated and analysed during the current study are not publicly available because they contain sensitive patient information, as all women are HIV-positive and suffering from perinatal depression, and their interviews cannot be de-identified. Furthermore, in our informed consent forms, it says, “We will not share the information you give us with anyone not involved in the study.” The ethics committee that imposes this is the National Health Sciences Research Committee in Malawi, as we used their informed consent forms. The committee can be reached at: Tel: + 265 1 726 422/418 or Email: mohdoccentre@nhsrc-mw.com. Structured interview guides are provided in English among the additional files. While the interview transcripts cannot be shared, the data matrices that summarize the interview content can be provided by the corresponding author.

## Abbreviations

HIV: Human Immunodeficiency Virus
PND: Perinatal Depression
WLHIV: Women Living with HIV
EPDS: Edinburgh Postnatal Depression Scale
LMIC: Low- and Middle-Income Countries
ART: Antiretroviral Treatment
mHealth: Mobile Health
SMS: Short Message Service
SRQ-20: Self-Reporting Questionnaire 20-item
