# Supplementary material for: The feasibility and acceptability of a task-shifted intervention for perinatal depression among women living with HIV in Malawi: a qualitative analysis
Source: BMC Psychiatry. 2022 Dec 29;22:833. doi: 10.1186/s12888-022-04476-z (PMC9798611; doi:10.1186/s12888-022-04476-z)
Supplement: Supplementary file 1 — Additional file 1. Perinatal Depression and HIV – Provider’s Interview Guide. [file 12888_2022_4476_MOESM1_ESM.pdf]

Perinatal Depression and HIV – Provider's Interview Guide

Overview (for interviewer): The goal of this in-depth interview guide is to explore HIV and mental health provider's perspectives on perinatal depression (PND), as well as staff resource needs, cultural appropriateness, and barriers and facilitators to PND screening and treatment.

Introduction: Hello, I am working on behalf of researchers at UNC Project-Malawi and we are very interested in your views about the following questions regarding nkhawa (stress, having worries) and kukhumudwa (depression):

1. Have you ever noticed women who are pregnant or recently had a baby having trouble with things that most people do not? For instance, trouble taking food, trouble engaging with their baby, or friends and family in a normal way (perhaps someone who isolates themselves)? Trouble doing household calls or even abusing substances to help cope with their problems? Possibly someone who is even thinking about harming themselves?
    a. How would you describe someone like this?
    b. Is there a particular word or set of words you would use to describe someone having those problems?

If no answer, further probe: What do you call it when someone is unhappy almost all of the time? What do you call it when this when it happens around the time of pregnancy?

2. The words we use as researchers to describe people with these issues might be something like nkhawa (stress, having worries) or even kukhumudwa (depression), have you heard those words before?
    a. If so, how have you heard them used?
    b. If not, how would you describe when women experience sadness or difficulty coping during pregnancy or the postpartum period?

3. We are very interested in hearing your opinion on women who experience kukhumudwa (depression) during pregnancy or after birth. What have you heard about women who experience kukhumudwa (depression)?
    a. How often do you see women experiencing kukhumudwa (depression) during pregnancy or after birth in your clinic?

4. Think back to the last time you had a pregnant or postpartum patient that you thought might be experiencing kukhumudwa (depression), tell me about them and their visit from the moment they came into your clinic.
    a. Probes:
        i. Tell me a little more about this patient?
        ii. What made you feel like they might be experiencing kukhumudwa (depression)?
        iii. What can you tell me about your interaction with this patient?
        iv. How did their possible kukhumudwa (depression) influence your clinical treatment of the patient?
5. How might someone experiencing kukhumudwa (depression) respond to HIV treatment?
        v. What would someone's kukhumudwa (depression) have to do with their ability to adhere to their HIV medications or attend their HIV visits?

        vi. In what other ways do you think being on HIV treatment might be different for someone who is experiencing kukhumudwa?

6. What treatments do you think would be best to address someone's kukhumudwa (depression)?

7. How often do you think you see patients that are experiencing kukhumudwa (depression)?
    a. Are there situations during pregnancy or the postpartum period that you see these patients more frequently?
        i. Probe: what about when a woman is diagnosed with HIV, after a woman miscarries, or gets pregnant without a husband.

Many women who have HIV also experience kukhumudwa (depression). Because of this, we are interested in exploring the possibility of screening for as well as treat kukhumudwa (depression) in antenatal and HIV clinics like this one.

8. For women who are pregnant, how do you think being diagnosed with HIV during pregnancy affects whether or not they develop kukhumudwa (depression)?
    a. In your experience, are women with HIV more or less likely to develop kukhumudwa (depression) during pregnancy?

9. If a woman experiences kukhumudwa (depression), how do you think it may affect her engagement in her HIV care?
    a. From your clinical experience, how has kukhumudwa (depression) affected women's ability to take their HIV medications? What about attending their HIV appointments?

10. If there were a survey that had about ten questions where a patient's total score told you whether or not a patient has kukhumudwa (depression) (and how severe it might be), how realistic would it be for you to integrate a questionnaire like that into your normal work as a clinician?
    b. How do you think most providers would think about that?
    c. Who would be the best person in your clinic be to complete the survey with each patient?

11. Some patients who experience kukhumudwa (depression) can be effectively treated by speaking with a trained counselor, others can be treated well through taking medications, what have you heard about these methods of treatment?
    b. Views on counseling:
        i. What types of women with kukhumudwa (depression) do you think would benefit the most from counseling?
            1. Probe: all women, HIV-infected women
        ii. What do you think the best way to deliver counseling would be in your clinic? Who would be the best person to deliver counseling in your clinic? What should the counseling sessions cover?
        iii. Do you think it would be better for women to talk to counselors individually or with other women also experiencing depression?
    c. Views on medication:
        i. What types of women with kukhumudwa (depression) do you think would benefit the most from medication?
            1. Probe: all women, HIV-infected women

d. If services like these were available to you as a clinician, what do you think would be the best way to deliver them to women with kukhumudwa (depression)?
    i. Who would deliver the services, when should they be delivered, where should the services be delivered?
        1. Probe: religious counselors, trained healthcare providers
e. Views on training:
    i. What sort of additional training would be necessary for you and your colleagues to treat patients with depression using either medication or counseling?
    ii. What sort of additional supervision would be necessary for you and your colleagues to treat patients with depression using either medication or counseling?
    iii. How do you think that supervision for treating kukhumudwa (depression) might be best delivered to you and your colleagues?
